# Why do we need improved mobility technology?

**DOI:** 10.1186/1743-0003-9-16

**Published:** 2012-03-30

**Authors:** Michael L Boninger, Rachel E Cowan

**Affiliations:** 1 Professor and Chair, Department of Physical Medicine and Rehabilitation, University of Pittsburgh, Pittsburgh, PA, USA; 2 Professor, Departments of Bioengineering and Rehabilitation Science and Technology, University of Pittsburgh, Pittsburgh, PA, USA; 3 Medical Director, Human Engineering Research Laboratories, VA Pittsburgh Healthcare System, Pittsburgh, PA, USA; 4 3471 Fifth Avenue, Suite 201 Pittsburgh, PA 15213 USA; 5 Post-Doctoral Associate, The Miami Project to Cure Paralysis, Miller School of Medicine, University of Miami, Miami, FL, USA; 6 Lois Pope Life Center, 1095 NW 14th Terrace, Miami, FL 33136 USA

## Introduction

The National Science Foundation (NSF) in collaboration with the World Technology Evaluation Center (WTEC) decided to make inclusion of scientists with disabilities a priority when selecting the investigators for a trip investigating research conducted in Europe on mobility technology for people with disabilities. The trip was rigorous with groups visiting multiple labs and multiple countries often in a single day. A typical day began with a 7 or 8 am checkout, a cab ride to the first location, followed by cabs to two or three more locations, all the while toting luggage. After the daily tours were complete, yet another cab ride to the airport or train station, travel to a new city, cab to the hotel, check in, hunt down dinner, and with any luck, in bed by 11 pm. This was the pattern for five days, a taxing schedule for any individual, disabled or not. The rigorous travel and obstacles encountered further emphasized the need for this study. The purpose of this paper is to highlight the obstacles our group faced during our travels as concrete example of how mobility limitations can impede participation.

### Imagine

Imagine you want to take a vacation to Europe, you will visit great cities and eat wonderful food. Not hard to picture. The image is fun, unencumbered by worries of transportation, accessibility, and discrimination. Okay maybe there are some worries, but you have done this before. Now imagine you have a disability that requires you to use a wheelchair.

### Air Travel

You will be the first person on each flight and last person off, adding an hour to the trips you plan to take by plane. Maybe it is not so bad hanging out in an airport, they have shopping now, right? Not for you, you will be segregated; forced to wait in special holding areas. When you do get to leave the holding area, it will not be to the local coffees shop in the terminal, you will be the first person on the plane; there when they are still cleaning up from the last flight. Why do you have to be early? Because you have to be loaded onto a very narrow chair and dragged down the aisle in order to get to your seat. You'd be blocking traffic if you loaded at the same time as everyone else. If you are lucky, there is a jet way or mechanical lift to get you to the aircraft door. If you are unlucky, you get the added fun of being dragged up the stairs. Have a seat, relax, they have just taken your wheelchair; placed it on the tarmac and will load it into the belly of the plane after the luggage goes in (you hope). If they lose that piece of valuable luggage you are stranded. I hope you went to the restroom before you got on, and didn't drink much fluid all day, because you can't walk to the restroom, they took your wheelchair (which wouldn't fit down the aisle anyway). The extra transfers, four just to get on a plane, are starting to wear on your shoulders. One person must accompany you for this odyssey, you can't travel alone. Have more than one friend, too bad, someone gets to hang out in the terminal alone.

### Trains

The trains allow the avoidance of security, but at least with security there is someone to help. The train station is a different story. You book on a train that is listed as being accessible. It is not. You don't know this until the train arrives and you are faced with two choices, miss the train and figure out a different way to get to the next town, or be physically lifted onto the train, about 5 steps. When you get on the train, putting your luggage up is impossible on your own. Again traveling alone is not recommended. So you bring a friend, who is just there to help you get on the train, not to travel. Unfortunately, while trying to get you situated, the train starts to move; he is stuck on the train and has to pay for a round trip fare to a location he did not want to visit. At least you have him for the other scary part, trying to get off.

### Oh the Places You Won't Go

The other small matter from the trip is all the places you can't see - the wonderfully inaccessible churches, bars, restaurants, and research labs. Yes, research labs meant to study ways to improve the lives of individuals with disabilities. The planned walk across campus that is nearly impossible because of uneven pavement or steep hills, to a building where you have to go in the back door because it is the only entrance with a ramp. It has been a long day, the hotel room calls you. An accessible room? Perhaps. Then there is you night time routine, which takes longer than your non-disabled peers. Your day, already lengthened by the extra time at the airport, just got a bit longer. All the time, the one constant is the need to ask others for help.

## Discussion

Everything discussed above happened on our trip every day. Many other inconveniences, scary moments, and inaccessible locations are not included. Could we have avoided this with great planning? Some of it, but not most. This is not the fault of the person with a disability. His or her ability to travel as freely as the rest of the group is restricted because society has not taken the steps to ensure equal access. Without the kindness of strangers and friends, the trip would not have been possible and many of the obstacles could not have been surmounted.

### Imagine

Now imagine that the goals of all the researchers we met have been achieved. Movement, the way you interact with the environment, is not restricted. Either technology has allowed for further healing, you have technology that replaces the function you have lost, or society has removed the barriers. Either way it's a great trip.

